# Postpartum-Specific Anxiety and Maternal–Infant Bonding: A Predictive Validity Study amongst Italian Women

**DOI:** 10.3390/ejihpe14060107

**Published:** 2024-06-04

**Authors:** Chiara Ionio, Giulia Ciuffo, Paul Christiansen, Anna Maria Della Vedova, Victoria Fallon, Maria Francesca Figlino, Marta Landoni, Sergio A. Silverio, Martina Smorti, Alessandra Bramante

**Affiliations:** 1 CRIdee, Unità di Ricerca sul Trauma, Dipartimento di Psicologia, Facoltà di Psicologia, Università Cattolica del Sacro Cuore, 20123 Milan, Italy; giulia.ciuffo@unicatt.it; 2 Department of Psychology, Institute of Population Health, Faculty of Health and Life Sciences, University of Liverpool, Liverpool L69 3GF, UK; paul.christiansen@liverpool.ac.uk (P.C.); vfallon@liverpool.ac.uk (V.F.); sergio.silverio@kcl.ac.uk (S.A.S.); 3 Dipartimento di Scienze Cliniche e Sperimentali, Facoltà di Medicina e Chirurgia, Università Degli Studi di Brescia, 25123 Brescia, Italy; anna.dellavedova@unibs.it; 4 Dipartimento di Psicologia, Facoltà di Psicologia, Università Cattolica del Sacro Cuore, 20123 Milan, Italy; mariafrancesca.figlino@unicatt.it; 5 CRIdee, Dipartimento di Psicologia, Facoltà di Psicologia, Università Cattolica del Sacro Cuore, 20123 Milan, Italy; marta.landoni@unicatt.it; 6 Department of Women & Children’s Health, School of Life Course & Population Sciences, Faculty of Life Sciences & Medicine, King’s College London, London WC2R 2LS, UK; 7 Dipartimento di Patologia Chirurgica, Medica, Molecolare e dell’Area Critica, Università di Pisa, 56126 Pisa, Italy; martina.smorti@unipi.it; 8 Policentro Donna, 20123 Milan, Italy; alessandra.bramante@gmail.com

**Keywords:** anxiety, pregnancy, maternal–fetal attachment, postpartum bonding

## Abstract

The role of anxiety is unknown in relation to postpartum bonding, unlike the well-known detrimental effect that postpartum depression has on the relationship between a mother and child. This study investigates how anxiety affects mother–infant bonding after childbirth, comparing the Italian version of the Postpartum Specific Anxiety Scale (PSAS-IT) with generalized measures of anxiety. Examining 324 non-randomly-selected participants responding to various scales, including the Edinburgh postnatal depression scale (EPDS), generalized anxiety disorder (GAD), postpartum-specific anxiety scale (PSAS-IT), postpartum bonding questionnaire (PBQ), and baby care questionnaire (BCQ-2), initial results suggest a link between certain postpartum anxiety symptoms and attachment problems. Surprisingly, anxiety measured with the PSAS has no direct influence on attachment; however, it is a strong predictor of bonding, even when maternal age, general anxiety, and depression are taken into account, explaining 3% of the variance in scores (β = 0.26, *p* < 0.001). This emphasizes the importance of early identification and intervention of postpartum anxiety in promoting bonding between mother and child.

## 1. Introduction

Bonding between a mother and her child is inherently linked to a child’s survival and healthy future development [1]. Numerous studies have focused on the bonding between mother and child [1,2,3]. The mother–child bond is one of the most important psychological processes throughout the first postpartum year [3]. During interactions with their mother, newborns begin to internalize implicit social principles such as reciprocity, mutuality, and contingency [3].

Pregnancy is a crucial time for the mother–infant relationship to first establish [4,5,6]. Accordingly, the most important time for the development of a strong bond between a mother and her child is immediately after birth [5,6,7]. Whilst most women and their children develop a close relationship, some may find it difficult to form a strong emotional bond with their child [8]. Studies suggest inadequate mother–infant bonding occurs in 6–41% of mother–infant dyads [9,10]. During pregnancy and in the postpartum period, women are more prone to psychological problems such as increased stress, anxiety, and depression [10,11]. It is well known that depressive symptoms can affect the complex process of mother–infant bonding [12]. However, little is known about maternal bonding in women with postpartum anxiety disorders, despite the high incidence of anxiety disorders in the postpartum period, particularly in Italy [13]. Indeed, in August 2020, the Italian National Institute of Health conducted a multi-center study which found the prevalence of postpartum anxiety in Italy is more than double the overall prevalence of 15% (1 to 24 weeks postpartum) and 14.8% (>24 weeks) found in meta-analytical studies [13,14]. Despite increasing scientific evidence in favor of early detection and immediate treatment of maternal anxiety, in pregnant women in Italy, anxiety is still largely undiagnosed and untreated [13,14]. As a result, there is little research, particularly in Italy, on the prevalence and risk factors of anxiety in the postpartum period [15], and generic measures of anxiety are often used, which may have psychometric shortcomings and are not able to accurately capture unique presentations, as they can lead to false-positives and exaggerated anxiety scores [15]. The pregnancy anxiety scale (PAS) [16], the pregnancy-related anxiety questionnaire (PRAQ) [17], the revised PRAQ (PRAQ-R) [18], and the pregnancy-related anxiety scale (PRAS) [19] are just some of the self-report questionnaires which have been developed over time to address this gap. These questionnaires were developed to measure specific anxieties that relate solely to the antenatal period and not the postnatal period. The postpartum-specific anxiety scale (PSAS) is the only questionnaire specifically designed to measure postpartum anxiety, and has been developed in recent years by Fallon and colleagues [20] with the aim of developing and validating a measure which can accurately reflect the specific anxieties faced by postpartum women, thereby closing the existing methodological gap. According to the psychometric data collected by the researchers, the PSAS is a robust, credible, and acceptable study instrument for assessing postpartum worry and anxiety, which are underestimated in the literature [20].

Indeed, there are few studies on how mothers with postpartum anxiety disorders interact with their newborns. Some have found that newborns of mothers with anxiety disorders show higher stress reactivity during free play than newborns in the control group [3,21,22]. In addition, babies of mothers with anxiety disorders showed lower levels of social engagement compared to newborns of control groups, as evidenced by lower levels of attention, social initiation, vocalization, gaze maintenance, and good affect [3,21]. In addition, newborns of socially anxious mothers showed lower social responsiveness to strangers compared to controls [3,23]. It has also been found that exploratory play in children aged three to nine months was more pronounced in mothers with low anxiety [3,24].

There are other studies on maternal interaction, but the results are mixed. According to some research, mothers suffering from extreme anxiety may not be as empathetic with their newborns as controls [21,24,25], but other studies found no differences in this area [23]. In addition, women who suffered from severe anxiety often exhibited overly vigilant or anxious behavior in social situations, making them watchful and, in some cases, even withdrawn [26]. Other studies have linked anxious parenting to increased parental control (e.g., high intrusiveness and excessive regulation of child behavior) [27,28,29]. These findings are consistent with the observation that mothers with anxiety disorders according to the Diagnostic and Statistical Manual of Mental Disorder (DSM-IV) [30] allow their 10-year-old child less autonomy than mothers in good health [3]. When comparing anxious mothers with their healthy counterparts, the former showed less warmth and positivity and the latter more criticism and catastrophizing. Unfortunately, there are fewer studies on younger children, and it is unclear whether the maladaptive interaction patterns of anxious mothers are due to comorbid conditions, such as depression, or differences in the methodology of the studies [31].

Given the inconsistent findings on mother–infant attachment and the lack of studies investigating maternal attachment in women with anxiety, there is a definite need for research in this area. Given the high incidence of postpartum anxiety disorders and their potential impact on mother–infant interactions, research in this area is clearly warranted to prevent impairments in the relationship with their newborns and to promote and enhance mothers’ resources in terms of their bonding skills from an early stage.

This study aims to examine the predictive validity of the only assessment tool for postpartum-specific anxiety, the Italian-language version of the postpartum-specific anxiety scale (PSAS-IT) [14], to test whether postnatal maternal anxiety is a more effective predictor of maternal bonding and caregiving behavior than general measures of anxiety [32,33]. 

We hypothesize that, after controlling for the effects of generalized anxiety and depression, postpartum-specific anxiety will predict unique variance in maternal bonding scores. 

## 2. Methods 

### 2.1. Ethics

The study protocol was approved by the local (Tuscan) Ethics Committee, the ‘Comitato Etico Area Vasta Nord-Ovest’ (reference: CEAVNO N12749/2018), and by the research ethics committee of the University of Liverpool (reference: IPH/3964). 

### 2.2. Participants

Participants were recruited using (non-probability) opportunity sampling. This paper has followed the previous validation of the instrument from Ionio et al. 2023. Notably, an a priori power calculation based upon a small effect for the full model (f2 = 0.05) and the possibility of up 8 predictors (note that predictors beyond those specifically hypothesized were added based on testing associations, so the estimate is highly conservative) at 80% power with an alpha of 0.05 suggests that a minimum of 307 participants would be required. The inclusion criteria were strictly defined and included women over the age of 18 who were proficient in Italian, had no cognitive impairment, and had no history of psychiatric disorders. Exclusion criteria were applied to participants who reported having a mental illness and/or a traumatic event in the family within the last six months. In addition, people with psychotic disorders or posttraumatic stress disorder (PTSD) were excluded, in order to take into account the possible influence of clinically diagnosed and/or treated mental illness and PTSD on postpartum-anxiety symptoms.

The study cohort included mothers (*n* = 457) with newborns aged between birth and six months postpartum, with 324 participants included in the final analyses, and the others were removed due to missing data on the postpartum-specific anxiety scale (PSAS), the postpartum bonding questionnaire (PBQ), and/or the baby care questionnaire (BCQ). 

### 2.3. Measures 

Participants were asked to provide relevant personal information and were presented with a series of psychometric scales. The demographic data and psychometric instruments used in the study reflect the data routinely collected and used in the Italian healthcare system.

#### 2.3.1. Demographics

Participants’ demographic data included a range of variables, including age, ethnicity, place of birth, country of residence, occupation, educational level, and marital status. In addition, participants were asked about their mental health, with a focus on a clinical diagnosis of anxiety and depression. Demographic information about the child included details such as age, weight, length, multiple birth status, birth order, gestational age, and dietary habits. 

#### 2.3.2. The Edinburgh Postnatal Depression Scale (EPDS) 

The EPDS [34,35] is a self-report instrument consisting of 10 items designed to assess the extent of maternal depression in the postpartum period. Respondents must indicate the intensity of their feelings in the past week on a 4-point Likert scale. The total score of the EPDS ranges from 0 to 30. The validated Italian version of the EPDS has shown commendable validity and reliability, as evidenced by a Cronbach’s α coefficient of 0.7894, confirming the effectiveness of the instrument in detecting postnatal depression. The optimal threshold for clinically significant postnatal depression, determined with the EPDS in Italy, is in the range of 9 to 10 [36]. The questionnaire had good internal consistency (Cronbach’s α = 0.88). 

#### 2.3.3. The 7-Item Generalized Anxiety Disorder Scale (GAD-7)

The GAD-7 [37] is a short instrument consisting of 7 items and was developed for rapid screening of generalized anxiety disorder. Participants are asked to rate the extent to which they have suffered from anxiety in the last two weeks on a 4-point Likert scale. The total score of the GAD-7 ranges from 0 to 21. Previous evaluation of the instrument has shown that the optimal trade-off between sensitivity and specificity for diagnosing GAD was identified at a cut-off point of ≥10. The questionnaire showed good internal consistency (Cronbach’s α = 0.87).

#### 2.3.4. Postpartum Specific Anxiety Scale, Italian-Language Version (PSAS-IT)

The PSAS-IT [14] is a self-report instrument comprising 51 items designed to assess the frequency of specific anxiety symptoms during the postpartum period. Respondents are asked to rate their experiences over the past week on a 4-point Likert scale, ranging from 1 (never) to 4 (almost always). The total score on the PSAS ranges from 51 to 204, with a threshold score of 112, indicating a clinically significant level of anxiety. The PSAS was originally developed as an English-language tool [20] and contains these four factors: maternal competence and bonding anxieties (15 items), infant safety and welfare anxieties (11 items), practical infant care anxieties (7 items), and psychosocial adjustment to motherhood (18 items). To date, there have been two derivative short-form research studies [38,39], and the tool has been translated and validated in French [40], Spanish [41], Chinese [42], Persian [43], and Italian [14], the latter of which has been used in this study. The scale showed good internal consistency (Cronbach’s α = 0.94).

#### 2.3.5. Postpartum Bonding Questionnaire (PBQ)

The PBQ [44] is used to assess the quality of the parent–child bond and to recognize possible disturbances in the relationship. It has proven to be particularly valuable in identifying challenges that can impair the mother–child bond. The questionnaire consists of 25 items spread across four scales, and includes impaired bonding (12 items), rejection and pathological anger (7 items), anxiety for the child and care (4 items), and imminent abuse or risk of abuse (2 items). Each item within these scales characterizes an attitude that a parent may exhibit towards their child. Respondents, either mothers or fathers, are asked to honestly indicate how often they experience the feelings expressed in each item. This is determined using a 6-point Likert scale ranging from 0 (never) to 5 (always). The total achievable score is 125, with higher scores indicating greater bonding impairments. The authors suggest a threshold of 26, indicating the presence of a disorder, and 40, indicating the potential for a severe disorder. The following specific thresholds are also recommended for individual scales to identify potential difficulties: 12 for the impaired bonding scale, 17 for the rejection and pathological anger scale, 12 for the fear for the child and care scale, and 3 for the threat of maltreatment or danger of maltreatment scale. In terms of reliability, subsequent studies reported internal consistencies (Cronbach’s α) ranging from 0.76 to 0.87 for the overall scale [44]. The questionnaire had good internal consistency (Cronbach’s α = 0.91).

#### 2.3.6. Baby Care Questionnaire Version 2 (BCQ-2)

The BCQ, version 2 [45], is a self-report instrument for measuring parental beliefs about childcare and consists of 30 items divided into these three sections: sleeping (9 items), eating (10 items) and consoling (11 items). The items are rated on a 4-point Likert scale, ranging from strongly disagree (1) to strongly agree (4). The test can be carried out on the mother from the last trimester of pregnancy until the child is 18 months old. For each of these three caregiving contexts, the principles of structure (the extent to which parents endorse routine and regularity in childcare) and attunement (the extent to which parents endorse close physical contact and rely on the child’s signals) are measured by parents’ agreement with a series of statements, while practices are measured using checklists and quantitative questions (such as estimated duration). In terms of reliability, the questionnaire had good internal consistency (structure: Cronbach’s α = 0.87; attunement: Cronbach’s α = 0.83). 

### 2.4. Data Collection

Mothers of newborns aged between birth and six months postpartum were recruited via online advertisements with a link to the Qualtrics software (QualtricsXM 2024). The advertisements were disseminated in discussion forums for parents in Italy and on social media platforms. Each participant’s response was linked to a unique ID within the survey program to ensure anonymity. The link to the online survey remained active throughout the study period until the follow-up phase. An information sheet on the first page explained the aims and methods of the study. Participants had the option of withdrawing from the study at any time without giving a reason, as participation was completely voluntary. If they found themselves in distress or asked for support, they were referred to the local study leader, who referred them to a therapist. Participants’ contact details, which were collected at the end of the survey, were kept separate from the survey responses and processed anonymously, identified only by a unique ID number. The database was kept secure and was only accessible to members of the research team.

### 2.5. Statistical Analyses

SPSS statistics version 29.0 was used to analyze the data. First, a descriptive analysis was performed to examine the demographic characteristics of the sample. To examine the predictive validity of the PSAS in the context of maternal bonding and infant care, we conducted five hierarchical multiple regression analyses to examine the association between PSAS scores on maternal bonding using the summed score of the overall PBQ and each of its four subscales, respectively. In addition, two hierarchical multiple regression analyses were conducted to examine the association between PSAS scores and the parental beliefs regarding childcare using the summed score of each of its two subscales, respectively. Confounders significantly associated with both exposure and outcome at *p* < 0.1 level were included in the final regression models. Maternal age was included in the final regression models in cases that were associated with both exposure and outcome. In these cases, maternal age was to be entered in block one, with general measures of anxiety (total score of GAD) and maternal depression (total score of EPDS) in block two and the PSAS in block three. In cases where the maternal age did not correlate significantly, general measures of anxiety and maternal depression were to be entered in block one and the PSAS in block two. 

## 3. Results

### 3.1. Sample Characteristics

The age of the final sample of mothers (*n* = 324) ranged from 18 to 33 (M = 26.25; SD = 5.14). Participants were predominately women from Italy (97.2%), married (54.3%), in possession of a degree (45.7%), in administrative professions (23.8%), primiparous (69.8%), and with singleton pregnancies (99.1%). See Table 1 for full demographic details.

### 3.2. Postpartum Anxiety and Mother–Infant Bonding (Table 2) 

The final regression model predicted 20% of the variance in overall bonding (R^2^ = 0.20, F (4) = 21.12, *p* < 0.001). After controlling for maternal age, general anxiety, and maternal depression, the PSAS uniquely explained 3% of the variance in scores and was positively associated with overall bonding (β = 0.26, *p* < 0.001).

With respect to impaired bonding, the final regression model predicted 18% of the variance (R^2^ = 0.18, F (3) = 24.22, *p* < 0.001). After controlling for general anxiety and maternal depression, the PSAS uniquely explained 1% of the variance in scores and was positively associated with impaired bonding (β = 0.18, *p* = 0.01). The final regression model predicted 10% of the variance in rejection and pathological anger (R^2^ = 0.10, F (3) = 12.60 *p* < 0.001). After controlling for general anxiety and maternal depression, the PSAS uniquely explained 2% of the variance in scores and was positively associated with rejection and anger (β = 0.21, *p* = 0.006). 

Considering anxiety for childcare, the final regression model predicted 26% of the variance in anxiety (R^2^ = 0.26, F (4) = 30.46, *p* < 0.001). After controlling for maternal age, general anxiety, and maternal depression, the PSAS uniquely explained 7% of the variance in scores and was positively associated with anxiety for the child and care (β = 0.39, *p* < 0.001). Lastly, the final regression model predicted 8% of the variance in imminent abuse or risk of abuse (R^2^ = 0.08, F (4) = 7.812, *p* < 0.001). After controlling for maternal age, general anxiety, and maternal depression, the PSAS uniquely explained 1% of the variance in scores and was positively associated with imminent abuse or risk of abuse (β = 0.19, *p* = 0.01) (See Table 2). 

**Table 2:** Hierarchical regression analysis demonstrating postpartum-specific anxiety as a predictor of mother–infant bonding (total and subscales) and of parental beliefs regarding childcare (principles of structure and attunement) after controlling for general measures of maternal depression and anxiety.

	Cumulative	Simultaneous		
	R^2^-Change	F-Change	β	*p*
**Total mother–infant bonding** Step 1 Maternal age	0.01	F (1) = 4.82 *	−0.12	0.02
Step 2 EPDS	0.16	F (2) = 31.12 **	0.37	<0.001
GAD			0.04	0.58
Step 3				
PSAS	0.03	F (1) = 13.78 **	0.26	<0.001
**Impaired bonding** Step 1 EDPS	0.16	F (2) = 32.60 **	0.41	<0.001
GAD			−0.00	0.96
Step 2 PSAS	0.01	F (1) = 6.36 *	0.18	0.01
**Rejection and pathological anger** Step 1 EPDS	0.08	F (2) = 14.68 **	0.30	<0.001
GAD			−0.02	0.29
Step 2 PSAS	0.02	F (1) = 7.812 *	0.21	0.00
**Anxiety for the child and care** Step 1 Maternal age	0.01	F (1) = 4.763 *	−0.12	0.03
Step 2 EPDS	0.18	F (2) = 37.47 **	0.05	0.26
GAD			0.19	<0.001
Step 3 PSAS	0.07	F (1) = 32.98 **	0.39	<0.001
**Imminent Abuse or Risk of abuse** Step 1 Maternal age	0.03	F (1) = 12.44 **	−0.19	<0.001
Step 2 EPDS	0.03	F (2) = 5.82 *	0.13	0.09
GAD			0.05	0.47
Step 3 PSAS	0.01	F (1) = 6.37 *	0.19	0.01
**Principles of structure**				
Step 1 Maternal age	0.01	F (1) = 4.32 *	0.11	0.03
Step 2				
EPDS	0.00	F (2) = 1.27	0.12	0.14
GAD			−0.05	0.48
Step 3				
PSAS	0.00	F (1) = 0.67	0.06	0.41
**Principles of attunement **				
Step 1 EPDS	0.00	F (2) = 1.05	−0.12	0.15
GAD			−0.07	0.37
Step 2 PSAS	0.00	F (1) = 0.00	0.00	0.99

Bold type indicates significant β and *p* values; ** *p* < 0.001 * *p* < 0.05.

### 3.3. Postpartum Anxiety and Parental Beliefs Regarding Childcare (Table 2)

The final regression model predicted 2% of the variance in principles of structure (the extent to which parents approve of using routine and regularity in childcare) and was not significant (R^2^ = 0.02, F = 1.88, *p* = 0.11). On the other hand, the final regression model on the variance in principles of attunement (the extent to which parents approve of close physical contact and rely on the child’s signals) was not significant (R^2^ = 0.001, F = 1.88, *p* = 0.55).

## 4. Discussion

The aim of this study was to test the predictive validity of postpartum anxiety in relation to maternal bonding and childcare and to investigate whether the PSAS might be a more effective predictor of maternal bonding and childcare than general measures of anxiety or maternal depression. The results suggest that high levels of specific postpartum anxiety were associated with greater difficulties in developing maternal bonding with children. 

This result is partially coherent with previous results in the literature. Indeed, previous studies investigating the association between observed mother–infant bonding in the first postpartum year and maternal anxiety disorders have mostly been small, cross-sectional, and have produced mixed results. A few studies [3,46,47] found no evidence of differences in maternal sensitivity between mothers with anxiety disorders compared to mothers without anxiety disorders. Nonetheless, some studies have discovered correlative data. In contrast to control mothers (*n* = 30) who were not diagnosed with a disorder, a group of 30 mothers with a combination of major depressive disorder (MDD), obsessive-compulsive disorder (OCD), and anxiety disorder were described as distant and unresponsive to their 3-month-old infants in an early study by Weinberg and Tronick [48]. Similarly, Warren et al. [22] found lower sensitivity in mothers with anxiety (*n* = 25) compared to control mothers (*n* = 24) during mother–infant interactions 4–6 months postpartum. When interacting with their 6-month-old infants, Kaitz, et al. [49] observed that there were no discernible differences in bonding between mothers with a current anxiety disorder (*n* = 36) and healthy controls without a diagnosis (*n* = 59). However, they observed that anxious mothers responded more exaggeratedly than non-anxious mothers in terms of gaze, language, and the expression of positive affect. This is contrary to previous findings that anxious mothers are more likely to be uninvolved [23,50] However, postpartum anxiety, as assessed by the PSAS, does not appear to be associated with a mother’s ability to provide care for her child. Specifically, analyses of PBQ subscales revealed that postpartum anxiety explained unique variance in altered bonding, rejection, anger, child-focused anxiety, and the care and possibility of imminent abuse or risk of abuse. As expected, the PSAS explained unique variance in the data after checking for general anxiety and maternal depression. These results seem to corroborate the PSAS predictive validity analysis conducted in an English-speaking sample [33] and reinforce the notion that postpartum-specific anxiety is a distinct construct which results in unique effects on maternal and infant health and behavior outcomes, supporting specific postpartum-anxiety literature (e.g., [51]). Therefore, it is evident that postpartum anxiety is a specific and differentiated construct, within which lies the specific concerns regarding the care of children and mother–infant bonding.

Interestingly, previous research has found that perceptions of maternal–fetal bonding were predictive of postpartum maternal sensitivity, but future research is needed to clarify how perceptions of mother–infant bonding can affect maternal care [13]. Furthermore, it is important to note that anxiety scores measured through GAD were not effective predictors of maternal bonding, which casts further doubts about the suitability of the tool in postpartum populations [28]. Furthermore, maternal depression and maternal age were a significant predictor in the models, which excluded postpartum anxiety as a predictor of bonding. This underlines the importance of subclinical negative mood symptoms in new mothers for childhood outcomes, as highlighted previously in work which found that impaired bonding in mothers with anxiety disorder may be due to concomitant subclinical depression [52].

No study conducted so far has investigated how maternal age can influence the development of bonding between mother and child. However, the current results demonstrate that postpartum-specific anxiety predicts impaired bonding independently of depressive symptoms and of maternal age. It is also true that the relationship between PSAS scores and the PBQ subscale of infant-focused anxiety is unsurprising, given that the PSAS focuses on anxieties specific to the infant.

Compared to our research on the associations between postpartum maternal anxiety and maternal childcare using BCQ subscales, postpartum anxiety does not appear to account for the variance in scores associated with mothers’ beliefs about caring for their children, suggesting postpartum-specific anxiety does not significantly influence parents’ endorsement of routine and regularity in childcare or close physical contact and reliance on infant cues. Even the measures of maternal depression and generalized anxiety do not predict the care of the child in our analyses. These results do not seem in line with the existing literature, because, although there are no studies specifically investigating parental approval of routine and regularity in childcare or of close physical contact and dependence on child signals, some studies have shown a strong association between maternal postpartum anxiety and dysfunctional behavior of mothers compared to the care of their children. For example, anxious parenting has been associated with increased parental control, an excessive regulation of the child’s behavior, and high intrusiveness [53]. In line with these findings, mothers with an anxiety disorder granted less autonomy to their 10-year-old child than healthy mothers, showed less warmth, and were more critical than their healthy counterparts [54]. It should be noted, however, that the study considered the relationships of mothers with children who were of school age. Unfortunately, research with younger children is limited. However, we would like to point out that a possible reading of our results could have a cultural origin, which was well investigated by a recent study by Ciuffo et al. [55]. Mothers with anxiety symptoms may not withdraw from the practice of caring for their children, because they may find it difficult to disengage from the social expectation of being the “good mother”. The study also found that the idea of “good parenting” tends to be even more restricted in the early stages of parenthood. In Italy, the dominant norm refers to the ideal of the ‘perfect mother’ who is available and attentive to the child’s needs in this period of high dependency and intensive care in which parents must respond to the child’s needs for nurturing, sleep, and emotional attachment [56]. Further studies are needed that can corroborate or discuss our findings.

Additionally, the association between postpartum-specific anxiety and infant feeding behaviors has been demonstrated previously [33] and is an important aspect to consider when discussing childcare. In particular, their results have shown that postpartum anxiety is significantly associated with lower chances of exclusive breastfeeding and breastfeeding in any amount in the first six months of life. Instead, our results have not shown such a significant impact of anxiety on care behavior, so it is important that future studies consider the need to investigate the role that postpartum maternal anxiety plays on childcare. In our model, the only significant, predictive variable compared to the extent to which parents approve of using routine and regularity in childcare (principles of structure) was maternal age. The literature suggests that older mothers are more inclined to adopt traditional parental models than their younger counterparts [57]. Furthermore, chronological age can have an impact on mothers’ affective behaviors [18,58], suggesting age at first birth appears to be positively associated with supportive maternal behaviors (e.g., physical affection). The PSAS work carried out to date has demonstrated the predictive validity of the tool with respect to infant feeding behaviors and mother–child bonding, but it did not produce significant results on the predictive validity of the tool compared to parents’ beliefs about childcare [32,59].

## 5. Strengths, Limitations, and Future Directions

In this investigation, data collection occurred online, implying a diminished degree of control over the sampling process. This study also had a self-selecting online sample consisting predominately of primiparous mothers. The generalizability in diverse samples, particularly those at risk of postpartum mental health problems, is necessary. The exclusion of women with a history of mental illness limits the generalizability of the results to non-clinical contexts. Furthermore, recruitment for this study took place during the COVID-19 pandemic, which could affect the results. However, our study clearly highlights the need to use specific measures to assess the anxiety of postpartum mothers, giving further evidence of the decreased effectiveness of the most-used tools nowadays. Additionally, our findings support the theory which sees a distinction between postpartum-specific anxiety and general anxiety disorder, providing further evidence of how postpartum anxiety may be a distinct construct, incorporated in the emotional and physical context of the months following childbirth with a new child, which can produce adverse outcomes on the development of the bond between parent and child. Indeed, one of the strengths of this study is its consideration of postpartum anxiety as a predictor of the development of the mother–child bond, which provides a more comprehensive overview of the strong relationship between maternal psychological health and healthy child development. Future research should seek to assess the role postpartum maternal anxiety plays on mothers’ beliefs about childcare and how such a psychopathological construct can foster adverse outcomes on bonding. Indeed, knowing the link between a mother’s mental health and her behavior towards her child, as well as her attitude towards attachment, can help mothers to receive different therapies to prevent possible negative outcomes. Therefore, to prevent maternal mental illness from being unrecognized and untreated, intensive awareness campaigns and health services need to be put in place. Strategies to prevent postpartum disorders include teaching mothers how to interact with their infants in a way that strengthens the mother–infant bond, helping mothers find the best coping mechanisms for the situations and emotions they experience during this time, and having reasonable expectations for childbirth and parenthood.

## 6. Conclusions

Postpartum anxiety is associated with difficulties in maternal bonding. The PSAS-IT has been shown to be a clear and effective predictor of maternal–infant attachment. Understanding the nuanced relationships between postpartum anxiety, maternal–infant attachment, and parental beliefs about childcare is critical for targeted interventions. These findings underscore the need to address postpartum anxiety in comprehensive maternal and childcare support programs.

## Figures and Tables

**Table 1:** Characteristic of the study sample (*n* = 324).

Maternal Characteristic	Value
**Maternal age (mean years ± SD)**	26.25 ± 5.14
**Country of Residence (*n*/%)**	
Italy	315 (97.2)
UK	1 (0.3)
Germany	1 (0.3)
Spain	2 (0.6)
Other European	4 (1.2)
**Marital Status (*n*/%)**	
Married	176 (54.3)
Co-habiting	146 (45.1)
Single	1 (0.3)
Separated	1 (0.3)
**Occupation (*n*/%)**	
Managers, Directors, Senior Officials	14 (4.3)
Professionals	68 (21.0)
Associate Professionals and Technical	17 (5.2)
Administrative and Secretarial	77 (23.8)
Skilled Trade	53 (16.4)
Caring, Leisure and Other Service	24 (7.4)
Sales and Customer Service	30 (9.3)
Process, Plant and Machine Operatives	24 (7.4)
Housewife	17 (5.2)
Not in Paid Occupation	3 (0.9)
Unemployed	10 (3.1)
**Educational Attainment (*n*/%)**	
Middle school diploma	13 (4.0)
Secondary school	113 (34.9)
Undergraduate education	148 (45.7)
Postgraduate education	34 (10.5)
Other qualification	16 (4.9)
**Birth order (*n*/%)**	
1st	226 (69.8)
2nd	78 (24.1)
3rd	17 (5.2)
4th	2 (0.6)
5th or more	1 (0.3)
**Multiple birth (*n*/%)**	
Yes	3 (0.9)
No	321 (99.1)

## Data Availability

The raw data supporting the conclusions of this article will be made available by the authors, without undue reservation.
